# Receptor Tyrosine Kinase Signaling Involves *Echinococcus*–Host Intercommunication: A Potential Therapeutic Target in Hepatic Echinococcosis

**DOI:** 10.3390/tropicalmed9080175

**Published:** 2024-08-07

**Authors:** Haijun Gao, Zhuoma Bianba, Xiaojin Mo, Wei Hu, Zheng Feng, Fangye Zhou, Ting Zhang

**Affiliations:** 1 Chengdu Fifth People’s Hospital (Affiliated Fifth People’s Hospital of Chengdu University of Traditional Chinese Medicine; The Second Clinical Medical College), Chengdu 611130, China; ganzrcdc_ghj@163.com; 2 National Institute of Parasitic Diseases, Chinese Center for Disease Control and Prevention (Chinese Center for Tropical Diseases Research); National Key Laboratory of Intelligent Tracking and Forecasting for Infectious Diseases; National Health Commission Key Laboratory of Parasite and Vector Biology; WHO Collaborating Center for Tropical Diseases; National Center for International Research on Tropical Diseases, Shanghai 200025, China; moxj@nipd.chinacdc.cn (X.M.); huw@fudan.edu.cn (W.H.); zfeng0909@163.com (Z.F.); 3 National Health Commission Key Laboratory of Echinococcosis Prevention and Control, Xizang Center for Disease Control and Prevention, Lhasa 850000, China; bianzhen4210@126.com; 4 State Key Laboratory of Reproductive Regulation & Breeding of Grassland Livestock, School of Life Sciences, Inner Mongolia University, Hohhot 010070, China

**Keywords:** drug target, echinococcosis, *Echinococcus* metacestodes, receptor tyrosine kinase, tyrosine kinase inhibitor

## Abstract

Echinococcosis, one of the most serious and life-threatening parasitic forms of zoonosis worldwide, is caused by the larvae of *Echinococcus granulosus* (*E. granulosus*) and *Echinococcus multilocularis* (*E. multilocularis*). Various drugs are being applied clinically to treat zoonosis; however, their therapeutic efficacy remains a great challenge, especially with albendazole as the preferred drug of choice. Receptor tyrosine kinase (RTK) signaling controls normal cellular proliferation, differentiation, and metabolism in humans and mammals, which are intermediate hosts of *E. granulosus* and *E. multilocularis*. Disruption of RTK signaling can cause various forms of carcinogenesis and exacerbate the progression of certain forms of parasitic disease. As a result, a significant number of studies on tyrosine kinase inhibitors (TKIs) have been conducted for the treatment of cancer and parasitic infection, with some TKIs already approved for clinical use for cancer. Notably, RTK signaling has been identified in the parasites *E. granulosus* and *E. multilocularis*; however, the mechanisms of RTK signaling response in *Echinococcus*–host intercommunication are not fully understood. Thus, understanding the RTK signaling response in *Echinococcus*–host intercommunication and the potential effect of RTK signaling is crucial for identifying new drug targets for echinococcosis. The present review illustrates that RTK signaling in the host is over-activated following infection by *E. granulosus* or *E. multilocularis* and can further facilitate the development of metacestodes in vitro. In addition, some TKIs exert strong parasitostatic effects on *E. granulosus* or *E. multilocularis,* both in vitro and/or in vivo, through downregulation of RTK signaling molecules. The summarized findings suggest that RTK signaling may be a promising drug target and that TKIs could be potential anti-*Echinococcus* drugs warranting further research.

## 1. Introduction

Echinococcosis, a parasitic form of zoonosis, is caused by the larval stage of the tapeworm of the genus *Echinococcus* [1,2]. The two main types of the disease prevalent in humans are cystic echinococcosis (CE), caused by *Echinococcus granulosus* (*E. granulosus*), and alveolar echinococcosis (AE), caused by *Echinococcus multilocularis* (*E. multilocularis*), which pose a substantial threat to public health globally [3,4]. Of these two prevalent forms, CE has a global distribution, while AE is predominantly distributed in the cooler and temperate latitudes of the northern hemisphere [4,5,6,7], particularly in the Qinghai–Tibetan Plateau area of China [8,9]. AE causes a more significant economic and public health burden for humans than CE due to the cancer-like invasive growth manner of *E. multilocularis* metacestodes [10,11]. Upon infection, the parasite larvae reside most commonly in the liver of humans and mammals, with the latter serving as intermediate hosts in transmission [12,13]. Growing metacestodes lead to liver fibrosis and cirrhosis in the host, particularly *E. multilocularis* metacestodes, which are fatal if left untreated or inadequately treated because of their cancer-like invasive growth manner, earning them the title “parasitic cancer” [14,15]. At present, the main options for the treatment of liver echinococcosis include drug treatment, surgical resection, and liver transplantation (mainly for AE patients) [16,17]. Among the available chemotherapies, albendazole (ABZ), a benzimidazole derivative, is the preferred drug of choice [18,19]. However, the effectiveness of ABZ, the severe adverse effects caused by long-term application, and clinical recurrence remain significant challenges [18,19]. Thus, finding new drug targets and therapeutic agents is urgently required.

Receptor tyrosine kinase (RTK)-mediated signaling regulates essential cellular physiological processes, such as cell proliferation and migration, glucose uptake, and energy metabolism in humans and mammals [20,21], with the latter often acting as the intermediate host of *E. granulosus* and *E. multilocularis*. Published data show that the disruption of RTK signaling can cause various forms of carcinogenesis and promote cancer progression [22,23], indicating RTK signaling as a potential and promising therapeutic target in cancer. More importantly, an increasing number of tyrosine kinase inhibitors (TKIs) are in development for the treatment of cancer [23,24,25], such as linifanib, targeting vascular endothelial growth factor (VEGF), which induces excessive angiogenesis in solid cancers; gefitinib and cetuximab, targeting epidermal growth factor receptor (EGFR) signaling in non-small cell lung cancer and metastatic colon cancer; and some that act on chronic diseases, such as imatinib, targeting platelet-derived growth factor (PDGF) signaling in pulmonary hypertension and respiratory dysfunction. More importantly, some of these TKIs have been approved for the clinical treatment of certain types of cancer [23,24,25]. Thus, RTK signaling is a potential and promising therapeutic target for the treatment of cancer and other forms of chronic disease. Interestingly, RTK signaling has also been found to play an important regulatory role in the progression of many forms of parasitic diseases, such as schistosomiasis [26,27] and echinococcosis [28,29]. For example, a study by Brehm and Koziol demonstrates that the activation of RTK signaling can facilitate the development of *E. granulosus* germinative cells and protoscoleces [30]. In comparison, in other studies, some TKIs have been shown to inhibit *E. multilocularis* metacestode development in vitro and/or in vivo [31,32,33]. Herein, we summarize and discuss the recent studies (from January 2001 to April 2024) focusing on the RTK signaling response in the host after *Echinococcus* infection, the role of RTK signaling in *E. granulosus* and *E. multilocularis* metacestode development, and its anti-echinococcal effect in vitro and in vivo to provide information referring to potential drug targets for echinococcosis.

## 2. RTK Signaling in Humans and Mammals

RTKs, a family of evolutionarily conserved transmembrane proteins, govern cellular pathological processes in humans and mammals [20], such as sheep, goats, cattle, camels, mice, and pikas, which are intermediate hosts of *E. granulosus* and *E. multilocularis* larvae [13]. In these hosts, the RTK family contains a variety of essential receptors, such as EGFR, fibroblast growth factor receptor (FGFR), VEGF receptor (VEGFR), platelet-derived growth factor receptor (PDGFR), insulin receptor (IR) or insulin-like growth factor receptor (IGFR), hepatocyte growth factor receptor (HGFR or C-Met), and ephrin receptor (Ephr) [34,35]. As is widely acknowledged, distinct RTK classes can recognize different growth factors and hormone ligands, which include EGF, fibroblast growth factors (FGFs), VEGF, PDGF, insulin and insulin-like growth factor (IGF), hepatocyte growth factor (HGF), and nerve growth factor (NGF) [24,35]. Interestingly, different receptors in RTK signaling show some common structural characteristics, including an extracellular ligand-binding region (ELR), a membrane-spanning helix, and a tyrosine kinase-containing intracellular region [36]. In general, each of these receptors carries catalytic kinases that remain inactive as monomers but are promptly activated when the ELR binds to a specific ligand that is a soluble polypeptide, small-molecule protein, or hormone [35,36]. Once the ligand–receptor conjugation forms, leading to dimerization or oligomerization, it facilitates trans-autophosphorylation and relieves autoinhibition of the intracellular tyrosine kinase domain, promoting cell growth and proliferation by initiating downstream signaling cascades [37,38,39], such as Src homology-2 (SH2), mitogen-activated protein kinases/protein kinase B (MAPK/Akt), phosphoinositide 3-kinase (PI3K)/mammalian target of rapamycin (mTOR), and c-Jun N-terminal kinase (JNK).

## 3. RTK Signaling Identification in *E. granulosus* and *E. multilocularis*

It is recognized that genome-wide analyses have shown that RTK signaling is widely present in many parasite species, such as *Trypanosoma cruzi* [40], *Toxoplasma gondii* [41], *Plasmodium falciparum* [42], *Schistosoma* [43,44], and even *E. granulosus* and *E. multilocularis* [28,45] and the model invertebrate organism *Caenorhabditis elegans* [46]. In *E. granulosus* and *E. multilocularis*, some encoding genes for not only growth factor ligands but also their receptors in RTK signaling, such as EGFR and EGF, FGFR, IGF receptor (IGFR, e.g., EmIR1 and EmIR2), and insulin-like ligands (e.g., EmILP1 and EmILP2), excluding FGF, PDGF, PDGFR, VEGF, and VEGFR, have been identified [28,45,47] (Table 1). Interestingly, *E. granulosus* and *E. multilocularis* show a high degree of sequence homology with the receptors involved in RTK signaling that are derived from humans and mammals [28,45]. Furthermore, the sequence analyses indicate that these receptors and ligands in the RTK family have a high degree of similarity within the conserved motifs between the parasite and its intermediate hosts, respectively [28,45,48].

The results of the above studies indicate that RTK signaling may play important roles in *Echinococcus*–host intercommunication, although the detailed molecular mechanisms underlying the activation of RTK signaling in *Echinococcus* metacestode growth remain unclear. Further discussion should be conducted to provide clues as to the development of new anti-echinococcal drugs targeting RTK signaling.

## 4. RTK Signaling Response in *Echinococcus* Infected Hosts

Following *Echinococcus* metacestode infection, the host liver, as the primary organ of infection [49], undergoes a chronic, continuous, and gradual damaging progression, mainly exhibiting liver fibrosis and cirrhosis [50,51]. Simultaneously, the host shows a significant response in RTK signaling after infection of *E. granulosus* and *E. multilocularis* larvae [50,52,53]. For example, in *Echinococcus*-infected mice, a significant increase in VEGF mRNA/protein expression was observed in the liver around the parasite metacestodes, accompanied by a rise in VEGF content in the serum [52,54,55]. In studies conducted by our research group, excessive VEGF-induced pathological angiogenesis was found to occur in the liver around the parasite metacestodes in mice following intraperitoneal infection with *E. multilocularis* metacestodes [52,56]. However, whether this phenomenon was caused by excessive expression of VEGF and VEGFR in the infected hosts remains unclear and thus necessitates further investigation.

Insulin, a regulatory molecule involved in RTK signaling, has been studied extensively in humans and mammals, with high concentrations mainly found in the liver [30]. Beyond this, insulin signaling has been studied in *Caenorhabditis elegans* and *Drosophila melanogaster* [57,58] because in the two model organisms, cell metabolic processes, growth, proliferation, and reproduction are controlled by conserved insulin signaling. Interestingly, insulin signaling has been shown to play an important role in many helminths, such as *Schistosoma japonicum* and *Schistosoma mansoni* [59,60], in addition to *Echinococcus* spp. [30,61]. Organ tropism toward the host liver has been demonstrated in *E. granulosus* and *E. multilocularis* larvae [30,62]. In an in vitro study, human insulin showed a growth-promoting effect on *E. multilocularis* metacestodes in vitro, indicating that insulin or IGF-mediated signaling is closely related to *Echinococcus* metacestode growth [31]. However, how RTK signaling is involved in the host response to *E. granulosus* and *E. multilocularis* metacestode infection remains unclear.

Moreover, the expression of FGF was significantly increased in the host liver after infection with *E. granulosus* and *E. multilocularis* metacestodes [50]. Similarly, Förster’s study demonstrated that human FGF, which is widely expressed in the fibrotic liver but not in the normal liver, can stimulate the development of *E. granulosus* and *E. multilocularis* protoscoleces in vitro [32]. This finding indirectly indicates the over-activation of FGF signaling in the host after infection with the parasite; however, the response of FGF signaling following *E. granulosus* and *E. multilocularis* metacestode infection is not fully clarified.

Overall, *E. granulosus* and *E. multilocularis* metacestode infection can cause an excessive activation state of RTK signaling with a significant increase in growth factors in the parasite’s host. However, whether these growth factors in the infected host could promote *E. granulosus* and *E. multilocularis* metacestodes development remains unclear and thus necessitates further investigation.

## 5. Activation of RTK Signaling Involves *Echinococcus* Metacestode Development

Since human- or mammalian-derived growth factors or hormone ligands in RTK signaling have been found to promote the entry, survival, and replication of intracellular pathogens [63,64], an increasing number of investigators have begun to explore whether extracellular parasites can utilize these growth factors or hormones to maintain their survival and growth [32,33,65]. For example, Jin’s study showed that a putative EGFR-like kinase in *Toxoplasma Gondii* was activated under the stimuli of human EGF or rNcMIC3, which contains four EGF domains [64]. Similarly, in vitro, human EGF was shown to promote the growth and development of Planaria, which is a free-living cestode [66], and *Schistosoma mansoni* [43]. Therefore, understanding whether the ligand molecules in host RTK signaling could promote *E. granulosus* and *E. multilocularis* metacestode development is important for developing effective anti-echinococcal drugs.

The results of Feng’s study showed that EGFR signaling in *E. multilocularis* may be activated by human EGF in vitro, and human EGF could promote the development of *E. multilocularis* protoscoleces into microcysts [65]. Furthermore, evidence from in vitro studies indicates that the concentration of 10 ng/mL or higher of human recombinant EGF could significantly facilitate the growth and development of germinative cells of *E. multilocularis* metacestodes; in comparison, a physiological concentration of 1 ng/mL only exhibited a modest effect on *E. multilocularis* metacestode growth and development [33] (Table 2). This finding indicates that in humans and the intermediate host, under the stimuli of the physiological concentration of host EGF, the development of *E. multilocularis* metacestodes occurs over long periods spanning several years or decades, rather than as a swift or transitory process.

As is widely acknowledged, FGFR signaling, one of the conserved RTK signaling systems in humans and mammals, may be activated by FGF binding to FGFR, promoting cell homeostasis and persistent differentiation [63,67,68]. In *E. granulosus* and *E. multilocularis*, the FGFR encoding gene was identified through the use of high-throughput sequencing analysis; however, the FGF ligand was absent [28,45]. In Forster’s study, under the stimuli of different concentrations of mammalian FGF in vitro, ranging from 10 nM to 100 nM, the growth and development of *E. multilocularis* metacestode vesicles and primary cells were significantly boosted [32]. Notably, physiological concentrations of mammalian FGF lower than 10 nM showed only a moderate effect on the growth promotion of *E. multilocularis* metacestodes in vitro [32]. It is suggested that, with the support of physiological concentrations of host FGF, inapparent *E. multilocularis* metacestode infection in the infected host progresses for a longer duration.

IR/IGFR signaling is widely distributed in humans and mammals (e.g., rodents, artiodactyls, and Canidae) [69,70] and even in some parasites, such as *Schistosoma* as a helminth [59], and *E. granulosus* [63]. In IGF-R/IR signaling, there are three ligands (including IGF-I, IGF-II, and insulin) and three receptors (including IGF-IR, IGF-IIR, and insulin receptor) [70]. Evidently, IGFs, which are structurally and functionally similar to insulin, regulate longer-term glucose homeostasis by controlling insulin sensitivity [30,70]. Interestingly, the genes encoding IR (e.g., Em1 and Em2) and insulin-like ligands (e.g., EmILP1 and EmILP2) in *E. granulosus* and *E. multilocularis* show high structural and functional homology to those in humans and mammals (e.g., Canidae, artiodactyls, and rodents) [30]. Thus, we speculate that, in the infected host, IGFR/IR signaling in *E. granulosus* and *E. multilocularis* could be activated by host IGF and insulin. Furthermore, in vitro cultivation suggests that a continuous supply of glucose is crucial for nutrient uptake and energy metabolism in the parasite, depending on the activation of IGFR signaling supported by host-derived IGFs [61]. In summary, IGFR/IR signaling plays an important role in *Echinococcus*–host interaction and is a potential drug target for the treatment of liver echinococcosis in the future.

In both in vivo mouse models and humans, VEGF and VEGFR mRNA and/or protein levels in a number of studies were found to significantly increase following *Echinococcus* metacestode infection [52,56,71], indicating that abundant pathological angiogenesis or neovascularization in the liver around *E. granulosus* and *E. multilocularis* metacestodes may be caused by the excessive expression of VEGF and VEGFR. Angiogenesis is a crucial contributory factor in exacerbating liver fibrosis [72], which is the most typical process of *Echinococcus*–host intercommunication [31,73]. Thus, VEGF/VEGFR-induced angiogenesis is an important regulator in *Echinococcus*–host intercommunication; however, how the VEGF/VEGFR-induced angiogenesis promotes parasite growth and metastasis to other organs is not well understood.

HGF, a growth factor in RTK signaling secreted by stromal cells, can bind the specific receptor (c-Met) to regulate cellular proliferation and apoptosis, extracellular matrix invasion, and angiogenesis in the liver [74,75]. The dysregulation of the HGF/c-Met axis leads to the invasion and progression of solid cancers by initiating the downstream PI3K/Akt and p38/MAPK signaling cascades [76,77]. In addition, it has been demonstrated that the activation of HGF/c-Met signaling can not only boost the growth and development of *Plasmodium berghei* and *Plasmodium falciparum* [78,79] but also induce angiogenesis [80,81], which contributes to *E. granulosus* and *E. mutilocularis* metacestode development and metastasis [30,73]. However, the detailed role of HGF/c-Met signaling in *Echinococcus*–host interaction remains unclear and thus necessitates further investigation.

Overall, infection with *E. granulosus* and *E. multilocularis* metacestodes can cause liver fibrosis in humans and intermediate hosts, and the fibrotic liver often shows hyperactivation of RTK signaling, with excessive expression of growth factor ligands in RTK signaling. Simultaneously, the increased number of growth factors can promote *E. granulosus* and *E. mutilocularis* growth and development in vitro. Thus, in *Echinococcus*–host intercommunication, RTK signaling plays important roles in *E. granulosus* and *E. mutilocularis* development, implying that RTK signaling is an important and promising drug target for echinococcosis.

## 6. Targeting RTK Signaling Implies Potential Drug Target for Echinococcosis

The expression of growth factors or hormone ligands in RTK signaling is significantly increased in the host liver following infection caused by *E. granulosus* and *E. mutilocularis* larvae [82,83]. Host growth factors in RTK signaling can promote *E. granulosus* and *E. mutilocularis* metacestode growth and development in vitro [31,32,33], indicating that treatment of echinococcosis through the inhibition of RTK signaling is possible.

In Cheng’s study, EGFR inhibitors (BIBW2992 and CI-1033) and the MEK/ERK inhibitor (U0126) displayed strong inhibitory effects on the viability of *E. multilocularis* metacestode germinal cells in vitro [33]. Concurrently, BIBW2992 showed strong protoscolicidal activities for *E. multilocularis* metacestodes in the infected mice used in the study [33]. Furthermore, the results of Forster’s study demonstrate that BIBF 1120, a tyrosine kinase inhibitor, has a clear concentration-dependent parasiticidal effect on *E. multilocularis* metacestode vesicles in vitro by inhibiting the activity of three *Echinococcus*-derived FGF receptors [32]. Thus, we speculate that the excessive expression of EGF and FGF in the fibrotic liver caused by *E. granulosus* and *E. multilocularis* infection is indispensable for *E. granulosus* and *E. multilocularis* metacestode growth and development. This finding further suggests that EGFR and FGFR signaling are potential drug targets for the treatment of echinococcosis. However, further exploration of new methods for screening anti-*Echinococcus* drugs using EGFR and FGFR signaling as target molecules is required.

Additionally, in in vitro cultivation systems of *E. multilocularis* larvae, the addition of human insulin can promote the phosphorylation of two insulin receptor-like kinases (EmIR1 and EmIR2), which are mainly distributed in *Echinococcus*’s glycogen storage cells, thereby boosting the increase in glucose uptake in *E. multilocularis* metacestode germinal cells [31,63]. However, the insulin receptor inhibitor HNMPA(AM)3 was shown to prevent *E. multilocularis* germinal cells from developing into metacestodes by inhibiting insulin signaling in the parasite [31]. Moreover, data from Yuan’s study show that anacardic acid, a natural product isolated from Brazilian cashew nutshell liquid, inhibited *E. granulosus* and *E. multilocularis* metacestode development in vitro and in infected mice, accompanied by the suppression of angiogenesis in the liver around *E. multilocularis* metacestodes and the downregulation of the expression of VEGF in the mice [52]. This finding indicates that inhibiting excessive vascularization caused by *E. multilocularis* metacestode infection in the host liver for the treatment of echinococcosis seems feasible. Therefore, the results of Jiang’s study show that the tyrosine kinase inhibitor sunitinib not only damaged *E. multilocularis* metacestode vesicles in vitro but also inhibited the development of *E. multilocularis* metacestodes in mice [84], accompanied by the inhibition of pathological angiogenesis. More importantly, anti-*Echinococcus* trials involving more inhibitors of RTK signaling should be initiated in vitro and in vivo to prove the efficacy of screening anti-*Echinococcus* drugs for RTK signaling in the future.

Overall, many putative RTK signaling inhibitors have been shown to suppress the larval growth and development of *E. granulosus* and *E. multilocularis* in vitro and/or in vivo, accompanied by a significant decrease in the expression of RTK signaling molecules (Table 3). Thus, the results of such investigations support RTK signaling as a potential and important drug target for the treatment of echinococcosis, and RTK signaling inhibitors represent promising anti-echinococcal drugs. However, the clinical use of RTK signaling inhibitors in in vivo trials still requires further exploration, although the results of the majority of the published studies conducted in vitro support this finding.

## 7. Conclusions and Outlook

**Figure 1:**
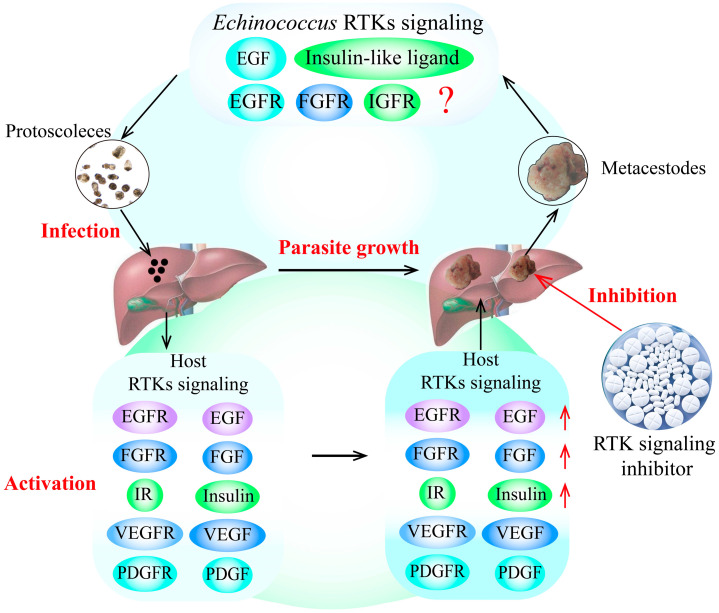
RTK signaling as a potential drug target for the treatment of echinococcosis. The findings indicate that the growth factors and hormone ligands involved in RTK signaling in the host are over-activated by *E. granulosus* and *E. multilocularis* infection. These over-expressed ligands can stimulate the growth and development of *E. granulosus* and *E. multilocularis* metacestodes by activating the specific receptors of RTK signaling in the parasite, indicating that RTK signaling may be an important drug target for the treatment of echinococcosis. In addition, the putative inhibitors of RTK signaling can block the development of *E. granulosus* and *E. multilocularis* metacestodes by decreasing the expression of RTK signaling molecules, indicating that TKIs are potential drugs for the treatment of echinococcosis. The red arrows indicates the upregulation of RTK siganling molecules expression, and the red question mark indictates that whether other host growth factors can combinate some *Echinococcus* receptors in RTK signaling to regulate *Echinococcus* spp. development remains unknown. Abbreviations: EGF, epidermal growth factor; FGF, fibroblast growth factor; IGF, insulin-like growth factor; IR, insulin receptor; PDGF, platelet-derived growth factor; RTKs, receptor tyrosine kinases; TKI, tyrosine kinase inhibitor; VEGF, vascular endothelial growth factor.

*E. granulosus* and *E. multilocularis* metacestode infection can cause excessive activation of RTK signaling pathways in the host, significantly increasing the expression of growth factors and hormone ligands. Furthermore, the over-expression of these growth factors and hormones in RTK signaling pathways in the host can stimulate the growth and development of *E. granulosus* and *E. multilocularis* metacestodes in vitro, possibly by activating the specific receptors of RTK signaling in the parasite, as the receptors from the parasite and its intermediate hosts have highly homologous protein sequences. Additionally, some putative RTK signaling pathway inhibitors block the growth and development of *E. granulosus* and *E. multilocularis* metacestodes, which is performed by downregulating RTK signaling pathways (Figure 1).

Therefore, RTK signaling plays an important contributory role in *Echinococcus*–host interactions, and it is an important drug target for echinococcosis. RTK signaling pathway inhibitors stand as promising future anti-echinococcal drugs. However, future efforts toward drug exploration for echinococcosis should focus on RTK signaling in vitro and in vivo. In addition, some lead compounds targeting RTK signaling need in-depth investigation before clinical trials are conducted.

## Figures and Tables

**Table 1:** Receptor tyrosine kinase signaling in *E. granulosus* and *E. multilocularis* and the parasite hosts.

Receptor	Ligand (Growth Factors and Hormones)	Receptor/Ligand in Humans and Mammals (the Parasite Host)	Receptor/Ligand in *E. granulosus* and *E. multilocularis*	References
EGFR	EGF	+/+	+/+	[28,33,45]
FGFRs	FGFs (FGF1 and FGF2)	+/+	+/−	[28,45]
PDGFR	PDGF	+/+	−/−	[28,45]
IR and IGF-Rs	Insulin and IGFs (IGF1 and IGF2)	+/+	+/+	[28,45,47]
VEGFRs (VEGFR1, VEGFR2, and VEGFR3)	VEGFs (VEGF1, VEGF2, VEGF3, VEGf4, and VEGF5), PIGF	+/+	−/−	[28,45]
HGFR or C-Met	HGF	+/+	−/−	[28,45]
Trk receptor	NGF	+/+	−/−	[28,45]

Abbreviations: *E. multilocularis*, *Echinococcus multilocularis*; *E. granulosus*, *Echinococcus granulosus*; EGF, epidermal growth factor; FGF, fibroblast growth factor; HGF, hepatocyte growth factor; IGF, insulin-like growth factor; IR, insulin receptor; NGF, nerve growth factor; PDGF, platelet-derived growth factor; PIGF, placental growth factor; PSC, protoscolece; RTKs, receptor tyrosine kinases; TKI, tyrosine kinase inhibitor; TRK, tropomyosin-related kinase; VEGF, vascular endothelial growth factor; +, present; −, absent.

**Table 2:** Activation of RTK signaling for *E. multilocularis* metacestode development by host growth factors in vitro.

Stimulus	Optimal Dose	Effects	Possible Mechanisms	References
Human EGF	100 ng/mL	Promotes MCs growth	Activating EGFR/EGF signaling	[33]
Human FGF	10 nM–100 nM	Promotes MCs proliferation	Activating FGFR/FGF signaling in *E. mutilocularis*	[32]
Human insulin	100 nM	Promotes GC, PSC, and MC development	Activating insulin/IR signaling	[31,61]

Abbreviations: *E. multilocularis*, *Echinococcus multilocularis*; *E. granulosus*, *Echinococcus granulosus*; EGF, epidermal growth factor; FGF, fibroblast growth factor; IGF, insulin-like growth factor; IR, insulin receptor; MCs, microcysts; PDGF, platelet-derived growth factor; PSC, protoscolece; RTKs, receptor tyrosine kinases; TKI, tyrosine kinase inhibitor; VEGF, vascular endothelial growth factor.

**Table 3:** TKR signaling inhibitors for the potential treatment of echinococcosis.

Compound	Structure	Parasite	Effects	Possible Mechanism	Reference
Nintedanib (BIBF1120)	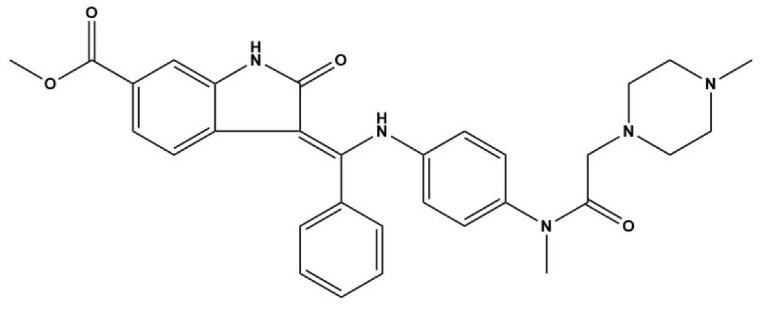	*E. multilocularis*	Inhibiting MC proliferation in vitro	Inhibiting FGFR/FGF signaling in *E. mutilocularis* in vitro	[32]
Afatinib (BIBW2992)	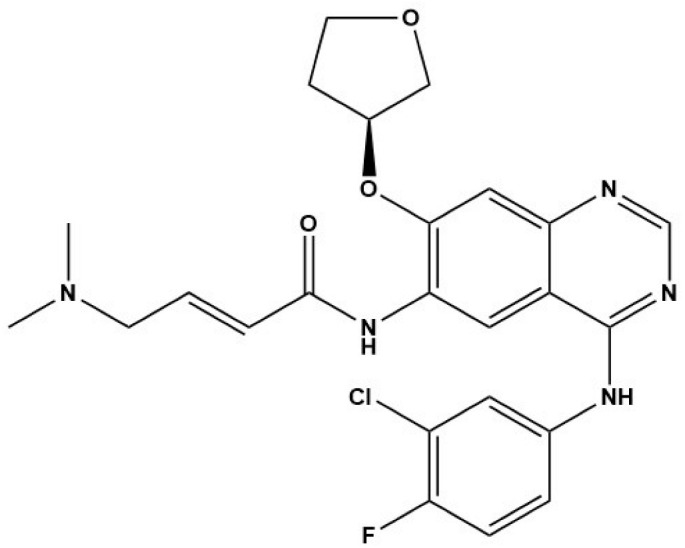	*E. multilocularis*	Inhibiting GC proliferation in vitro	Inhibiting FGFR/FGF signaling	[33]
Canertinib (CI1033)	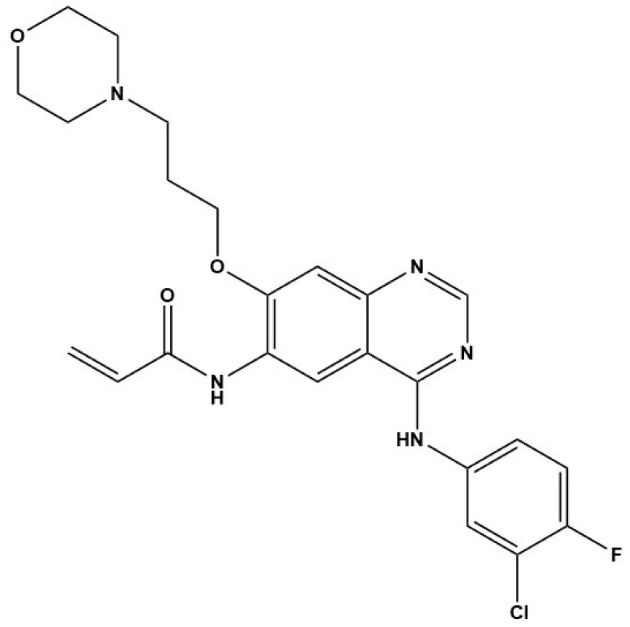	*E. multilocularis*	Inhibiting GC proliferation in vitro	Inhibiting FGFR/FGF signaling	[33]
HNMPA(AM)3	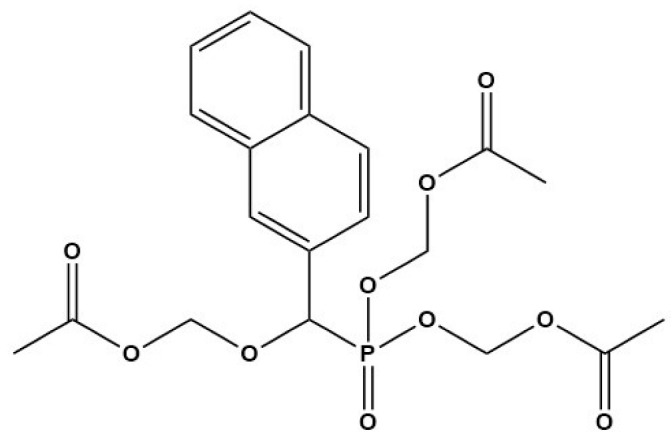	*E. multilocularis*	Inhibiting GC proliferation in vitro and decreasing the survival rate of PSCs and MCs in vitro	Inhibiting insulin receptor signaling	[31]
Anacardic acid	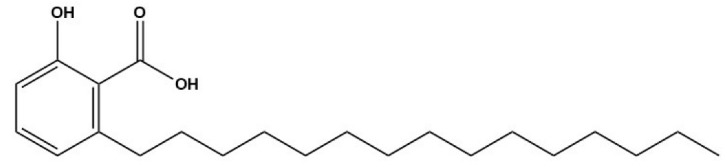	*E. multilocularis* and *E. granulosus*	Decreasing the survival rate of PSCs in vitro	Inhibition of VEGF-induced angiogenesis	[52]
Sunitinib	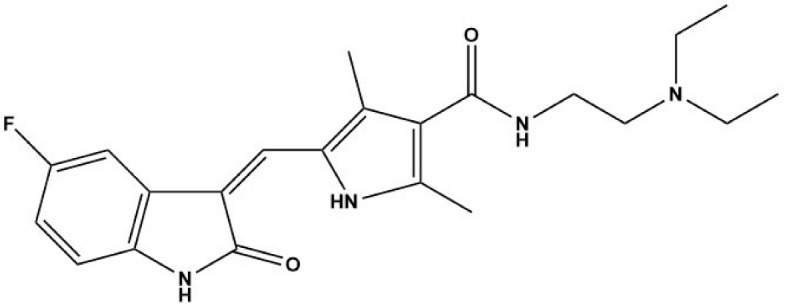	*E. multilocularis*	Inhibiting *E. multilocularis* metacestode vesicles in vitro and inhibiting *E. multilocularis* metacestodes in mice	Inhibiting VEGFA-induced angiogenesis	[84]

Abbreviations: EGF, epidermal growth factor; FGF, fibroblast growth factor; IGF, insulin-like growth factor; PDGF, platelet-derived growth factor; RTKs, receptor tyrosine kinases; TKI, tyrosine kinase inhibitor; VEGF, vascular endothelial growth factor.

## Abbreviations

ABZ, albendazole; AE, alveolar echinococcosis; AS, alternativesplicin; CE, cystic echinococcosis; *E. multilocularis*, *Echinococcus multilocularis*; *E. granulosus*, *Echinococcus granulosus*; EGF, epidermal growth factor; FGF, fibroblast growth factor; HGF, hepatocyte growth factor; IGF, insulin-like growth factor; IR, insulin receptor; MCs, microcysts; PDGF, platelet-derived growth factor; PSC, protoscolece; RTK, receptor tyrosine kinase; TKI, tyrosine kinase inhibitor; VEGF, vascular endothelial growth factor.
